# Constraint counting on RNA and ribosomal structures: linking flexibility and function

**DOI:** 10.1186/1758-2946-3-S1-O11

**Published:** 2011-04-19

**Authors:** Simone Fulle, Holger Gohlke

**Affiliations:** 1 Institute of Pharmaceutical and Medicinal Chemistry, HHU, Düsseldorf, Germany

## 

The ribosome is a large ribonucleoprotein complex that carries out protein synthesis in all kingdoms of life by translating genetic information encoded in mRNA into the amino acid sequence of a protein. The nascent polypeptides escape the peptidyl transferase center through the ribosomal exit tunnel that spans the entire large subunit. The tunnel is involved in the control of co-translational protein folding processes, the regulation of elongation and the inhibition of the protein synthesis by antibiotics [1]. Since the structure determination of the ribosome in atomic detail in 2000, much has been learned about the structural basis for protein synthesis. However, the functional role of the ribosomal exit tunnel has remained elusive and has been controversially discussed.

We thus set out to analyze global and local flexibility characteristics of the ribosomal exit tunnel by constraint counting on topological network representations of large ribosomal subunits from four different organisms [2,3]. The analyses provide critical insights into the role of the ribosomal exit tunnel during protein synthesis. The flexibility characteristics of the tunnel will be used to answer questions such as: What is the origin of species-selectivity of antibiotics binding? How is the co-translational elongation regulation regulated? What is the mechanism for signal transmission through the ribosomal structure?
